# Fatty acid profiling and oxidative stability of biscuits available in the market of the city of Ludhiana, India

**DOI:** 10.1038/s41598-023-44228-x

**Published:** 2023-12-08

**Authors:** Prabhjot Kaur, Monika Choudhary, Sanjula Sharma

**Affiliations:** 1 Department of Food and Nutrition, Punjab Agricultural University, Ludhiana, 141004 India; 2 Department of Plant Breeding and Genetics, Punjab Agricultural University, Ludhiana, 141004 India

**Keywords:** Biochemistry, Health care

## Abstract

India has occupied third position in biscuit manufacturing with an average production of 1.95 million tonnes. The major ingredients in biscuit manufacturing are refined wheat flour, sugar and fat. Fat to be used must be chosen carefully as it affects quality of final product in terms of fatty acid composition and oxidative stability. Therefore, the present work was planned to study fatty acid profile of highly consumed baked products of biscuit family such as biscuits and cookies available in market. The study was carried out to do fatty acid profiling of a range of highly consumed baked products of biscuit family as a primary objective and also, to determine oxidative stability of these products by analysing peroxide value and free fatty acid content. The most commonly consumed packaged and unpackaged bakery products were selected and were bought from the local market of Ludhiana city on the basis of a survey conducted on 200 subjects. The selected products were analysed for fatty acid composition and oxidative stability using standard methods. Fatty acid profiling of 22 bakery products of biscuit family was done. Palmitic acid was the most abundant among all fatty acids in packaged and unpackaged samples. Peroxide value of all the products even after storage period of three months was found below the permissible limits (< 10 meq/kg). Free fatty acids value of all the products also did not cross acceptable level of 0.5 percent. Out of total selected eight brands, six were national and two were international. Amount of palmitic acid was higher in the products belonging to local brands.

## Introduction

Bakery products are highly appreciated across the world. A wide range of bakery. products such as breads, biscuit, cookies and cakes etc. is available worldwide as per the demand of end users^2^. Biscuits are consumed on a daily basis by the consumers and have been categorized as fast-moving consumer goods (FMCG). Among FMCG category, the biscuit market is among the leading ones. The biscuit market reached $76.385 billion at the end of 2017 and expected to reach USD 164 billion by 2024 at compound annual growth rate (CAGR) of 5.08 percent^16^. The highest per capita consumption of biscuits in the world is approximately 13 kg per year^7^. In India, production of biscuits both in organized and unorganized sectors is estimated at 1.95 million tonnes per year^17^. The unorganized biscuit sector is estimated to have approximately 30,000 small and tiny bakeries across the country. Organized biscuit industry in India produces around 60 percent of the total production, the remaining 40 percent being contributed by the unorganized bakeries^3^. With these figures, India has occupied third position in biscuit manufacturing after USA and China^24^**.**

Baked products of the biscuit family are known variously as cookies, biscuits and crackers. The major ingredients are flour, sugar and shortening. According to Food Safety and Standards Authority of India (FSSAI), apart from maida (refined wheat flour) and sugar among major ingredients, biscuit formulation may contain vanaspati (partially hydrogenated vegetable oil) or refined edible oil, butter, ghee, margarine or their mixture with other minor ingredients (FSSA 2010). Food Safety and Standards Act of India specifies ash insoluble in dilute hydrochloric acid and acidity of extracted fat as quality parameters for biscuits. However, standards on nutrition quality of the fats used in the preparation of biscuits are not specified. Use of refined oils for the biscuit preparation will decrease the oxidative stability and provides poor texture and shorter shelf life^24,32^. The fats used in the manufacture of biscuits consist of both animal fat and vegetable shortenings with suitable solid fat index (SFI) and solid fat content (SFC)^31,35^**.** Different fats have been characterised based on its structural and physicochemical properties such as melting point, peroxide value etc. These properties further influence the final bakery product in terms of its sensory characteristics and shelf life. During heating or processing of fats, *cis*-unsaturated fatty acids are converted in to *trans* fatty acids (TFA) which have been associated with cardiovascular diseases^9^. Moreover, during storage, fats and oils are oxidized and free fatty acids are formed giving an unpleasant odour to the final product. So, this raises question about product quality^33^. Thus, fat to be used for the manufacturing of any bakery product must be chosen while giving full consideration to storage condition and desired shelf-life for the final product.

The label declaration of the commercial biscuits used in this study revealed the presence of either edible refined oil or hydrogenated oils as the fat ingredient. The partially hydrogenated vegetable oils (PHVO), also known as vanaspati is also being used in India for baking and frying process. Usually vanaspati is made up of vegetable oils and exist in the semi-solid form at room temperature^10,31^. Vanaspati (PHVO) provides up to 40 percent TFA and 30 percent saturated fatty acids (SFA). Consumption of TFA and SFA has been linked to an increase in the risk of cardiovascular disease. TFA raises LDL cholesterol, lowers HDL cholesterol, causes endothelial dysfunction and pro-inflammatory alterations, and may also cause insulin resistance and displace important fatty acids from membranes, increasing the risk of cardiovascular illnesses. As per the proposed regulation on TFA in partially hydrogenated vegetable oils (vanaspati) by FSSAI recommendation, TFA content should not exceed 10 percent of total fatty acids^10^. FSSAI has also recommended for mandatory labeling of TFA and SFA content of all edible oils and fats. FSSAI permits the health claims "trans-fat free" and "saturated fat free" to be made where the amount of trans fat in a serving of food is less than 0.2 g and 0.1 g, respectively^10^. So, there is a dire need for monitoring of the bakery products consumed on almost on daily basis in context with its fatty acid composition. Therefore, the present work has been planned to study the fatty acid profile of a range of highly consumed baked products of biscuit family such as biscuits and cookies available in the market.

## Methods

### Location of study

The current study was conducted in Ludhiana city of Punjab. Ludhiana is representative district of the state of Punjab as it is the most centrally located, the biggest and the most populous district in Punjab accounting for 12.6 percent of its population and also excels in the field of industries and agriculture. It also shares common boundaries with Rupnagar district in the east, Moga district in the west, and Barnala, Sangrur and Patiala districts in the south and southeast, respectively.

### Selection and preparation of samples

A survey was conducted on consumers’ behaviour in context with purchase and consumption of the bakery products in the Ludhiana city. For this, a total 200 subjects were selected using convenience sampling and was further be divided into two groups namely Youth (Age between 15 and 34 years) and Older Adults (Age between 35 and 60 years) having 100 subjects in each. An informed consent was obtained from the subjects to enrol them in the study. The data was collected just to have information regarding the category and type of bakery products consumed by the selected subjects.

On the basis of a survey, the most commonly consumed packaged and unpackaged bakery products were selected and were bought from the local market of Ludhiana city. Samples of the same product category/type, but from various producers, were analysed as separate samples. About 100–200 g of the food samples was grinded in mortar pestle. Further, the homogenized samples were stored in air tight polyethene pouches at 4 °C till further analysis. All methods for analysis were carried out in accordance with relevant guidelines and regulations. All experimental protocols were approved by the committee constituted under Dean, Post Graduate studies, Punjab Agricultural University, Ludhiana.

### Fatty acid profiling of selected bakery products

The Appelqvist (1971) method was used to prepare esters from oil samples for the fatty acid profiling procedure. Further 1 µl of methyl ester was injected at an 80:1 split ratio onto the FAME column CP-Sil 88, which has dimensions of 25 m × 0.25 mm × 0.20 mm and is fitted in the gas chromatography (GC) model 7820A series (Agilent technologies) connected to a flame ionization detector. The separation was completed at an oven temperature of 180 to 210 °C (rate of 4 °C per minute). The injector and detector were kept at 230 °C and 240 °C, respectively. Nitrogen, hydrogen, and air flowed at rates of 60-, 30-, and 30-ml min-1, respectively. For peak identification, standard fatty acyl esters' retention times (R) were used. Using EZ Chrome elite software, the relative concentration of each fatty acid was determined. The percentages of various fatty acids to the total fatty acids were used to express them.

### Oxidative stability of selected bakery products

The samples were kept at 37 °C for a period of three months. The oxidative stability in terms of peroxide value and free fatty acids of the samples were determined at 15 days intervals for 3 months using following methods.

#### Peroxide value

Peroxide value (PV) was determined using method given by AOAC (2000). For this, 5 g of sample was taken in the volumetric flask and then 50 ml of chloroform was added in to it. Volumetric flask was placed on shaker for 2–3 h for extraction of fat. Then, the extract was filtered with Whatman no. 1 filter paper. From filtered extract, 20 ml was taken in the flask and 30 ml of glacial acetic acid was added to it along with 1–2 ml of saturated potassium iodide solution. Then, the flask was left for 30 min. After 30 min, 50 ml of distilled water and 2 ml of 1 percent starch solution was added into the flask. It turned blue/black coloured solution. The solution was titrated against 0.01 N sodium thiosulphate until it turned to colourless solution. PV was calculated using formula as given below-

$${\text{PV }}\left( {{\text{meq}}./{\text{ kg}}} \right)~~ = \frac{{S \times N \times 1000}}{{{\text{Weight of sample}}}}$$

S = ml of 0.01N Na_2_S_2_O_3_ (Blank corrected used).

N = Normality of Na_2_S_2_O_3_.

#### Free fatty acids

Free fatty acids (FFA) were analysed using method given by Tarladgis et al. (1960). Five grams of sample was weighed and added to the flask containing 50 ml of benzene and kept for 30 min. After 30 min, the extract was filtered with the Whatman No. 1 filter paper. Then, 5 ml from the extract was taken in the flask and 5 ml of benzene, 10 ml of 95 percent ethanol and few drops of phenolphthalein indicator was added in the flask. The solution was titrated against 0.02 N potassium hydroxide till light pink colour appeared. FFA was calculated using formula as given below.

$${\text{FFA }}\left( \% \right)\, = \frac{{{\text{282}}\, \times \,0.0{\text{2N KOH}}\, \times \,{\text{ml }}\;{\text{of}}\;{\text{ alkali}}\;{\text{ used}}\, \times \,{\text{dilution }}\;{\text{factor}}\, \times \,{\text{1}}00}}{{{\text{1}}000\, \times \,{\text{weight }}\;{\text{of }}\;{\text{sample}}}}$$

### Statistical analysis

Mean and standard deviation for the various parameters were computed. One way ANOVA was applied for statistical analysis of data using data SPSS 26 (statistical package for the social sciences). To measure the difference between different treatments Tukey’s test (p < 0.05) was performed.

## Results

On the basis of survey, a total 22 samples including packaged and unpackaged bakery products were selected and purchased from the local market of Ludhiana, Punjab. The findings of the survey revealed that the majority of the respondents (50%) consumed 21.51 ± 0.23 g of biscuits once a day. Cookies were consumed by 60 percent of the respondents on weekly basis with an average intake of 27.71 ± 1.22 g. The selected samples then subjected to evaluation of fatty acid composition and parameters contributing to oxidative stability.

### Fatty acid profile of the selected samples

Among saturated fatty acids (SFA), Butyric acid (C4:0), Caprylic acid (C8:0), Decanoic acid (C10:0), Lauric acid (C12:0), Myristic acid (C14:0), Palmitic acid (C16:0), Stearic acid (C18:0) were analysed in the selected samples. Oleic acid (C18:1) and Linoleic acid (C18:2) were analysed among monounsaturated fatty acids (MUFA) and polyunsaturated fatty acids (PUFA), respectively.

**Figure 1:**
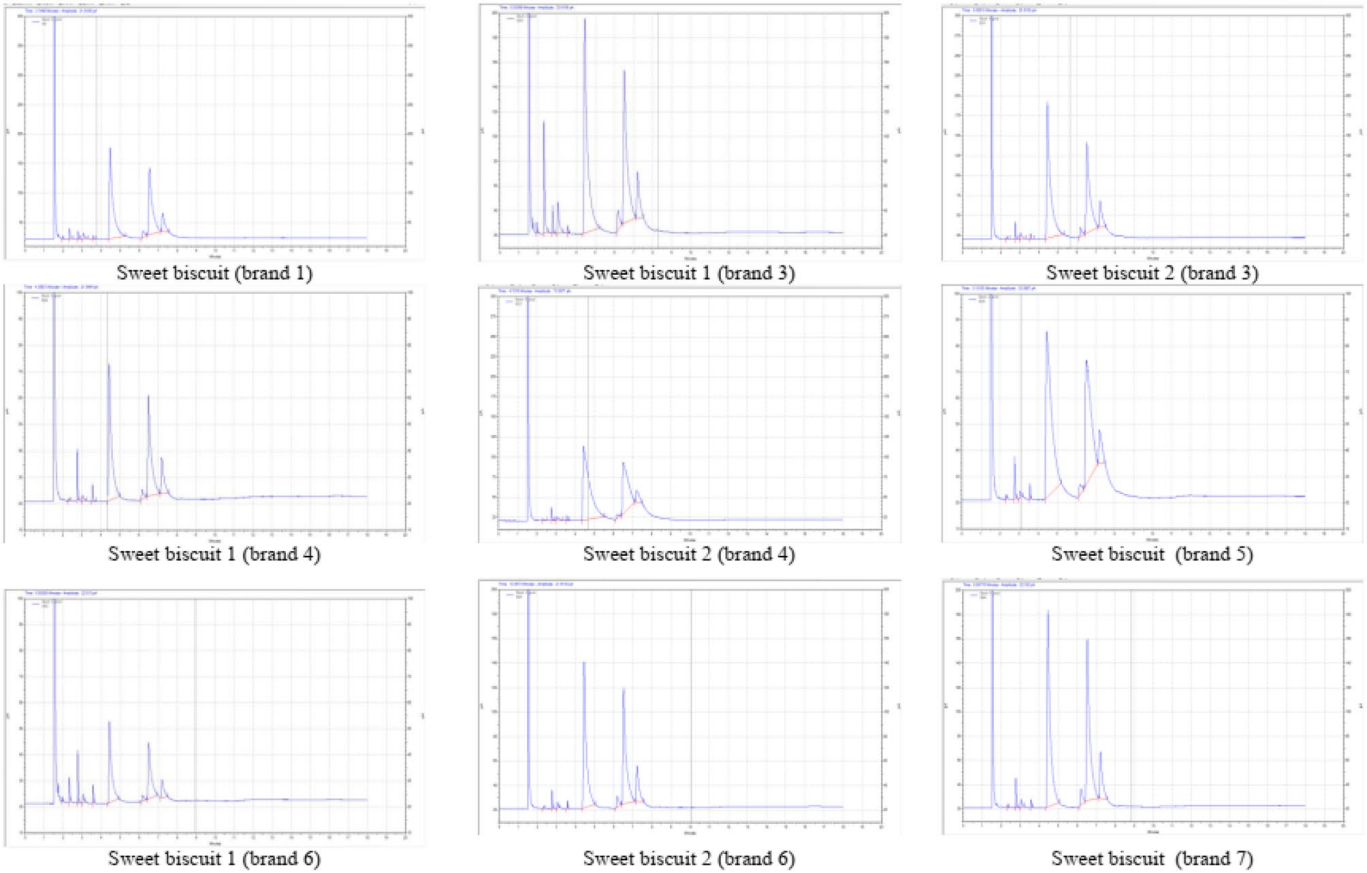
Fatty acids profile of sweet biscuits.

**Figure 2:**
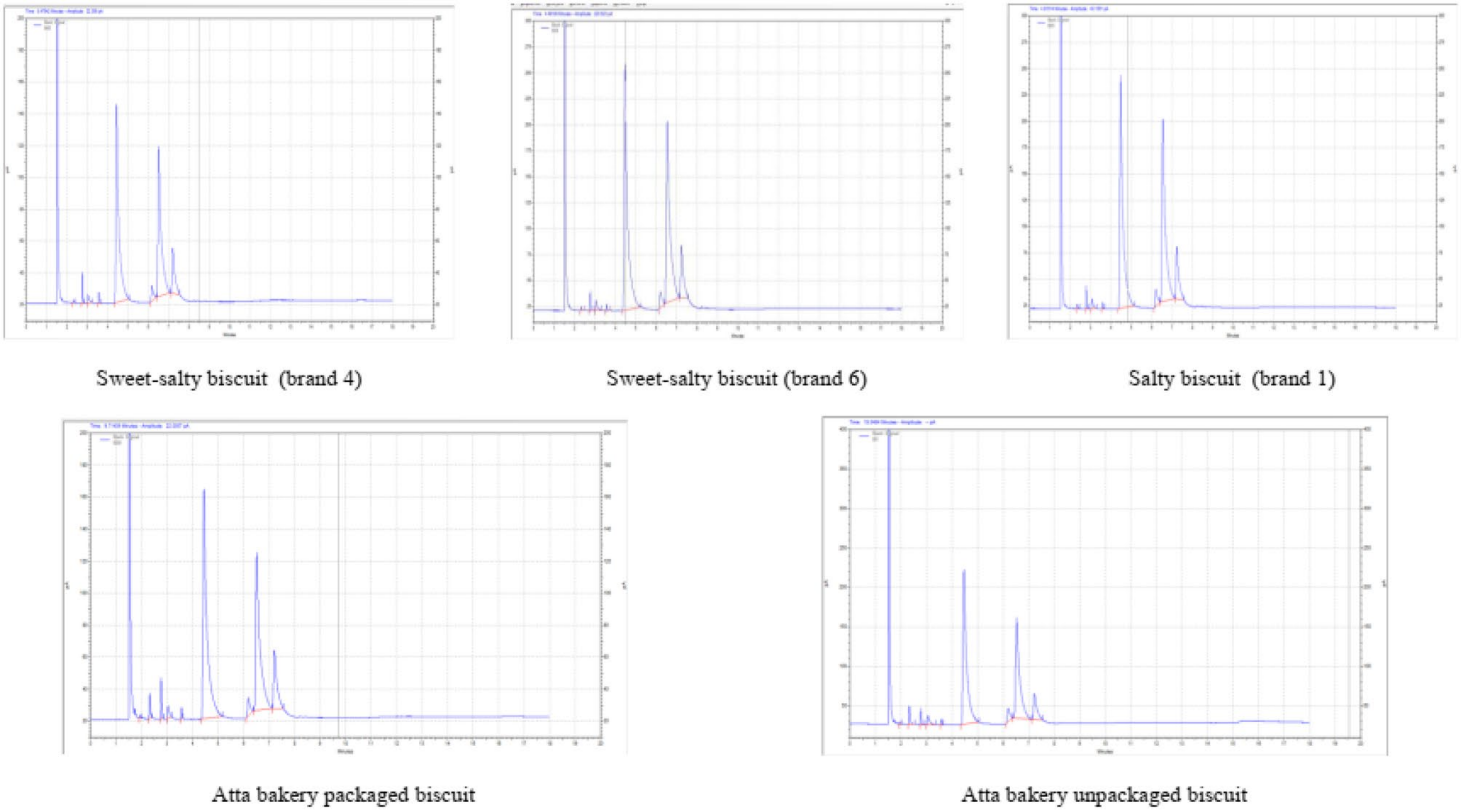
Fatty acid profile of sweet-salty, salty and atta bakery biscuits.

The fatty acid profile of biscuits is presented in Table 1. Palmitic acid was the major SFA found in all the biscuits. The range of palmitic acid in all the sweet biscuits lied between 42.95 ± 0.81 to 58.13 ± 1.68 percent. The corresponding figure in salty biscuits was recorded as 46.4 ± 0.29 percent. In sweet-salty biscuits, the palmitic acid was observed as 48.76 ± 0.92 percent in the biscuits of brand 6 and 46.38 ± 1.63 percent in biscuits of brand 4. The range of oleic and linoleic acid in sweet biscuits lied between 32.52 ± 0.29 to 39.44 ± 1.28 and 4.41 ± 0.02 to 10.49 ± 0.13 percent, respectively (Fig. 1). The corresponding figures in salty biscuits were 39.44 ± 1.28 and 9.23 ± 0.37 percent, respectively. In sweet-salty biscuits, the oleic acid and linoleic acid was observed as 38.34 ± 1.66 and 8.43 ± 0.19 percent in biscuits of brand 6 (Fig. 2). The corresponding figures in the biscuits of brand 4 were recorded as 38.22 ± 1.03 and 9.39 ± 0.14 percent, respectively. In packaged *atta* biscuit, the palmitic, oleic and linoleic acid were observed as 49.72 ± 2.21, 34.69 ± 1.23 and 7.52 ± 0.14 percent, respectively. *Atta* is an Indian name for whole wheat flour. The corresponding figures in unpackaged *atta* biscuits were 47.55 ± 1.97, 34.39 ± 1.15 and 10.32 ± 0.07 percent, respectively.

**Table 1:** Fatty acid profile of biscuits.

Bakery products	Fatty acid (%)								
	SFA							MUFA	PUFA
	Butyric acid (C4:0)	Caprylic acid (C8:0)	Decanoic acid (C10:0)	Lauric acid (C12:0)	Myristic acid (C14:0)	Palmitic acid (C16:0)	Stearic acid (C18:0)	Oleic acid (C18:1)	Linoleic acid (C18:2)
Sweet biscuit (Brand 1)	0.15 ± 0.00	1.52 ± 0.05	0.75 ± 0.03	1.53 ± 0.03	0.4 ± 0.01	49.15 ± 0.09	2.22 ± 0.09	36.62 ± 0.40	7.67 ± 0.18
Sweet biscuit 1 (Brand 3)	0.34 ± 0.01	6.34 ± 0.13	1.04 ± 0.04	3.23 ± 0.05	0.33 ± 0.00	45.49 ± 0.94	2.74 ± 0.00	33.31 ± 0.03	7.18 ± 0.30
Sweet biscuit 2 (Brand 3)	–	0.25 ± 0.01	1.13 ± 0.03	1.09 ± 0.04	0.4 ± 0.02	52.29 ± 0.66	2.14 ± 0.08	35.58 ± 0.87	7.14 ± 0.02
Sweet biscuit 1 (Brand 4)	–	0.25 ± 0.00	3.5 ± 0.01	0.92 ± 0.02	1.31 ± 0.03	44.48 ± 0.72	2.26 ± 0.01	36.8 ± 1.43	10.49 ± 0.13
Sweet biscuit 2 (Brand 4)	–	0.31 ± 0.01	1.24 ± 0.01	1.05 ± 0.00	0.4 ± 0.01	58.13 ± 1.68	1.18 ± 0.02	33.28 ± 1.35	4.41 ± 0.02
Sweet biscuit (Brand 5)	–	0.21 ± 0.01	1.69 ± 0.01	0.63 ± 0.02	0.55 ± 0.01	51.63 ± 0.98	1.42 ± 0.03	37.95 ± 0.31	5.92 ± 0.24
Sweet biscuit 1 (Brand 6)	0.54 ± 0.01	3.61 ± 0.12	5.75 ± 0.18	1.74 ± 0.04	2.08 ± 0.06	42.95 ± 0.8 1	2.4 ± 0.03	32.52 ± 0.29	8.41 ± 0.12
Sweet biscuit 2 (Brand 6)	–	0.15 ± 0.00	1.16 ± 0.04	0.75 ± 0.03	0.51 ± 0.01	45.71 ± 1.69	2.34 ± 0.05	39.39 ± 1.28	9.98 ± 0.10
Sweet biscuit (Brand 7)	–	0.17 ± 0.01	1.38 ± 0.01	0.8 ± 0.01	0.39 ± 0.02	44.94 ± 0.97	2.75 ± 0.01	40.12 ± 1.37	9.45 ± 0.06
Sweet-salty biscuit (Brand 4)	–	0.19 ± 0.01	1.55 ± 0.06	1.04 ± 0.05	0.6 ± 0.01	46.38 ± 1.63	2.63 ± 0.05	38.22 ± 1.03	9.39 ± 0.14
Sweet-salty biscuit (Brand 6)	–	0.2 ± 0.00	0.78 ± 0.00	1.01 ± 0.03	0.3 ± 0.01	48.76 ± 0.92	2.15 ± 0.05	38.34 ± 1.66	8.43 ± 0.19
Salty biscuit (Brand 1)	–	0.24 ± 0.01	0.97 ± 0.03	1.02 ± 0.04	0.3 ± 0.01	46.4 ± 0.29	2.41 ± 0.05	39.44 ± 1.28	9.23 ± 0.37
Atta bakery packaged biscuit	0.13 ± 0.01	1.81 ± 0.01	1.18 ± 0.03	1.64 ± 0.02	0.4 ± 0.01	49.72 ± 2.21	2.92 ± 0.12	34.69 ± 1.23	7.52 ± 0.14
Atta bakery unpackaged biscuit	0.15 ± 0.00	1.36 ± 0.03	1.72 ± 0.00	1.05 ± 0.00	0.49 ± 0.01	47.55 ± 1.97	2.48 ± 0.03	34.39 ± 1.15	10.32 ± 0.07

Values are mean ± SD, (*n* = 3). SFA = saturated fatty acids, MUFA = monounsaturated fatty acids and PUFA = polyunsaturated fatty acids.

**Figure 3:**
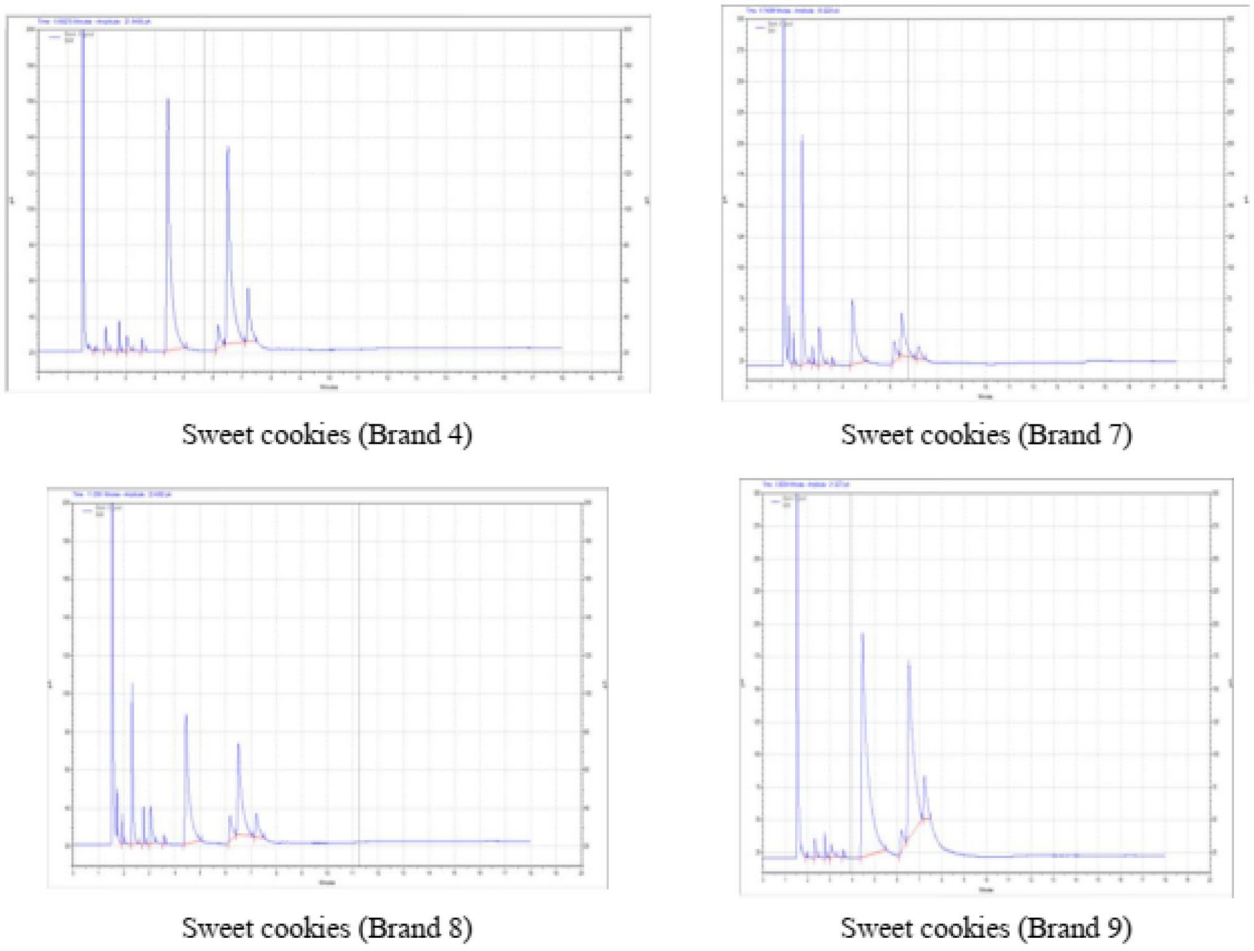
Fatty acid profile of sweet cookies.

**Figure 4:**
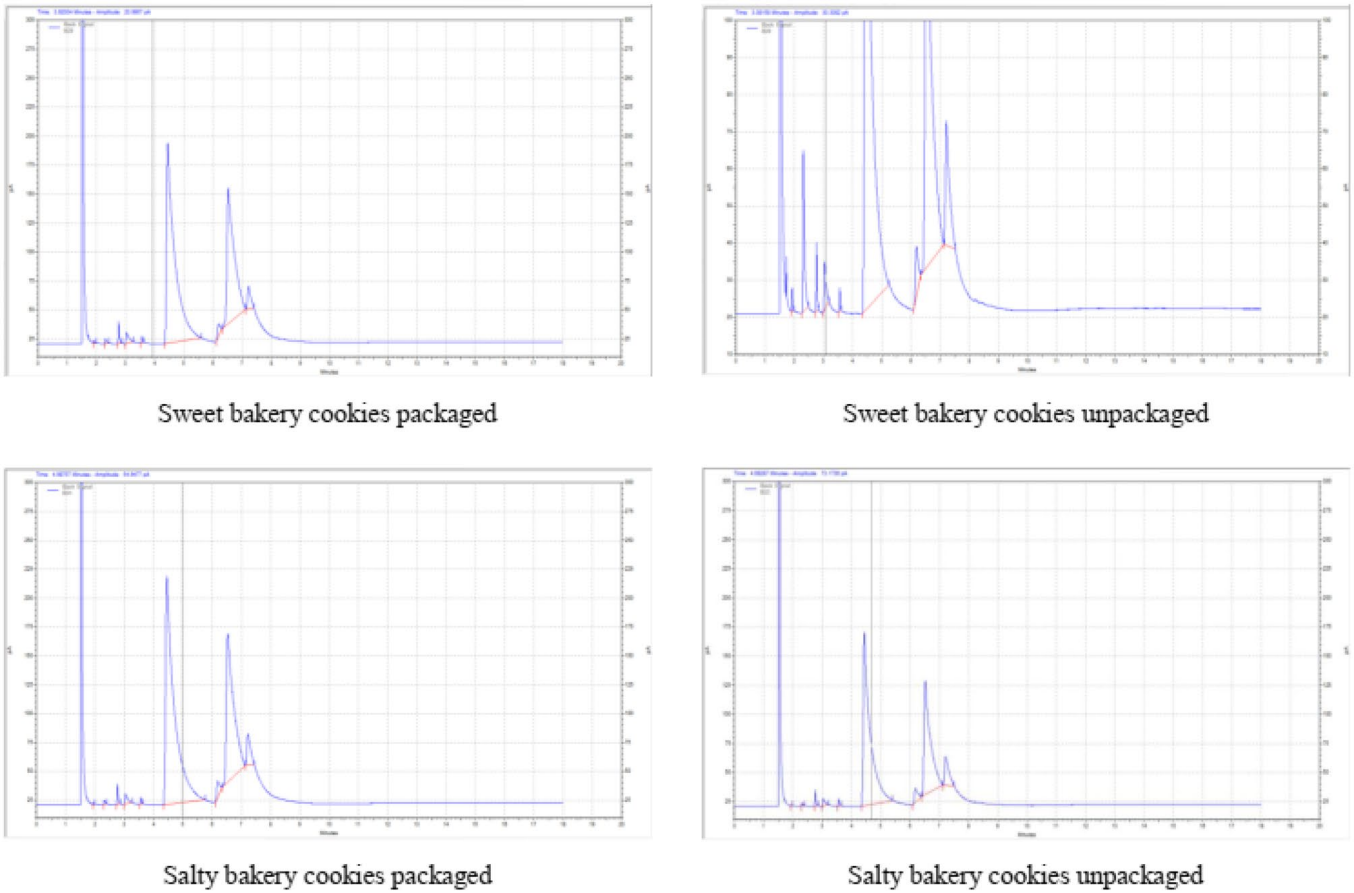
Fatty acid profile of bakery cookies.

Among SFA, palmitic acid was the majorly observed in all the cookies (Table 2). The range of average value of palmitic acid 28.37 ± 0.61 to 52.26 ± 1.04 percent, oleic acid 18.12 ± 0.57 to 39.01 ± 0.81 percent and linoleic acid 4.24 ± 0.00 to 8.31 ± 0.09 percent, were observed in sweet cookies from different brands (Fig. 3). In packaged salty cookies, the palmitic, oleic and linoleic acid were observed as 60.01 ± 1.89, 33.11 ± 0.27 and 3.63 ± 0.01 percent, respectively. The corresponding figures in unpackaged salty cookies were 55.46 ± 1.40, 34.22 ± 1.51 and 5.96 ± 0.06 percent, respectively (Fig. 4). In packaged sweet cookies, the palmitic, oleic and linoleic acid were observed as 58.33 ± 0.84, 35.31 ± 1.18 and 2.71 ± 0.06 percent, respectively. The corresponding figures in unpackaged sweet cookies were 52.14 ± 0.23, 34.4 ± 1.40 and 6.26 ± 0.17 percent, respectively.

**Table 2:** Fatty acid profile of cookies.

Bakery product	Fatty acid (%)								
	SFA							MUFA	PUFA
	Butyric acid (C4:0)	Caprylic acid (C8:0)	Decanoic acid (C10:0)	Lauric acid (C12:0)	Myristic acid (C14:0)	Palmitic acid (C16:0)	Stearic acid (C18:0)	Oleic acid (C18:1)	Linoleic acid (C18:2)
Sweet cookies (Brand 4)	0.18 ± 0.00	1.29 ± 0.03	1.21 ± 0.01	1.45 ± 0.01	0.57 ± 0.02	44.88 ± 0.16	3.15 ± 0.14	39.01 ± 0.81	8.31 ± 0.09
Sweet cookies (Brand 7)	2.32 ± 0.07	29.95 ± 0.24	1.5 ± 0.03	8.56 ± 0.05	0.69 ± 0.01	28.37 ± 0.61	6.25 ± 0.16	18.12 ± 0.57	4.24 ± 0.00
Sweet cookies (Brand 8)	1.51 ± 0.04	13.98 ± 0.60	2.22 ± 0.04	5.51 ± 0.18	0.59 ± 0.02	37.2 ± 0.74	5.38 ± 0.02	27.7 ± 0.17	5.19 ± 0.01
Sweet cookies (Brand 9)	0.1 ± 0.00	0.95 ± 0.01	0.81 ± 0.02	1.18 ± 0.04	0.24 ± 0.00	52.26 ± 1.04	2.35 ± 0.08	36.83 ± 0.40	5.29 ± 0.16
Sweet bakery packaged cookies	0.07 ± 0.00	0.27 ± 0.00	0.82 ± 0.02	1.12 ± 0.03	0.23 ± 0.01	58.33 ± 0.84	1.15 ± 0.03	35.31 ± 1.18	2.71 ± 0.06
Sweet bakery unpackaged cookies	0.22 ± 0.01	2.95 ± 0.05	0.77 ± 0.03	1.27 ± 0.01	0.26 ± 0.01	52.14 ± 0.23	1.73 ± 0.08	34.4 ± 1.40	6.26 ± 0.17
Salty bakery packaged cookies	0.02 ± 0.00	0.17 ± 0.00	0.64 ± 0.03	0.89 ± 0.00	0.19 ± 0.00	60.01 ± 1.89	1.35 ± 0.04	33.11 ± 0.27	3.63 ± 0.01
Salty bakery unpackaged cookies	0.02 ± 0.00	0.19 ± 0.01	0.73 ± 0.01	0.82 ± 0.00	0.34 ± 0.01	55.46 ± 1.40	2.26 ± 0.07	34.22 ± 1.51	5.96 ± 0.06

Values are mean ± SD, (*n* = 3). SFA = saturated fatty acids, MUFA = monounsaturated fatty acids and PUFA = polyunsaturated fatty acids.

### Oxidative stability of the selected bakery products

#### Peroxide value

The Table 3 presents the peroxide value of biscuits. The peroxide values in all the samples of biscuits increased significantly during storage. At initial stage, the peroxide value in sweet biscuits ranged between 0.45 ± 0.04 to 0.70 ± 0.07 meq/kg. The values in all the samples increased significantly during storage time. After 90 days, the corresponding figures ranged between 3.20 ± 0.00 to 4.89 ± 0.07 meq/kg. In terms of salty biscuits, the initial PV was recorded as 0.50 ± 0.07 meq/kg and after 90 days, PV reached to 3.80 ± 0.28 meq/kg. In sweet-salty biscuits, the initial PV in brand 4 was recorded as 0.69 ± 0.07 and after storage it increased to 3.86 ± 0.14 whereas the initial PV in brand 6 was observed as 0.65 ± 0.04 meq/kg and final PV was 3.70 ± 0.07 meq/kg. In packaged *atta* biscuit, the initial PV and final PV was observed as 0.70 ± 0.07 and 4.29 ± 0.07 meq/kg, respectively. The corresponding figures in unpackaged *atta* biscuits were 0.50 ± 0.07 and 3.44 ± 0.14 meq/kg, respectively. Therefore, PV of packaged *atta* biscuits was observed higher than PV of unpackaged *atta* biscuits.

**Table 3:** Peroxide value of biscuits.

Bakery products	Peroxide value (meq/kg)						
	0 DAY	15 DAY	30 DAY	45 DAY	60 DAY	75 DAY	90 DAY
Sweet biscuit (Brand 1)	0.70 ± 0.07^ g^	1.49 ± 0.14^f^	2.10 ± 0.07^e^	2.76 ± 0.28^d^	3.26 ± 0.14^c^	3.70 ± 0.07^b^	4.19 ± 0.28^a^
Sweet biscuit 1 (Brand 3)	0.45 ± 0.04^ g^	0.90 ± 0.07^f^	1.49 ± 0.07^e^	2.00 ± 0.28^d^	2.69 ± 0.07^c^	3.10 ± 0.14^b^	3.10 ± 0.14^a^
Sweet biscuit 2 (Brand 3)	0.59 ± 0.28^ g^	1.39 ± 0.00^f^	2.30 ± 0.07^e^	2.80 ± 0.28^d^	3.20 ± 0.00^c^	3.70 ± 0.07^b^	4.19 ± 0.00^a^
Sweet biscuit 1 (Brand 4)	0.55 ± 0.04^ g^	1.10 ± 0.21^f^	1.70 ± 0.07^e^	2.34 ± 0.04^d^	2.89 ± 0.07^c^	3.25 ± 0.04^b^	3.74 ± 0.04^a^
Sweet biscuit 2 (Brand 4)	0.50 ± 0.07^ g^	1.28 ± 0.14^f^	1.60 ± 0.00^e^	2.19 ± 0.14^d^	2.90 ± 0.07^c^	3.29 ± 0.07^b^	3.86 ± 0.14^a^
Sweet biscuit (Brand 5)	0.65 ± 0.04^ g^	1.30 ± 0.07^f^	1.88 ± 0.07^e^	2.39 ± 0.00^d^	3.00 ± 0.28^c^	3.60 ± 0.00^b^	4.09 ± 0.07^a^
Sweet biscuit 1 (Brand 6)	0.45 ± 0.04^ g^	0.90 ± 0.07^f^	1.50 ± 0.07^e^	1.90 ± 0.07^d^	2.40 ± 0.14^c^	2.70 ± 0.14^b^	3.20 ± 0.00^a^
Sweet biscuit 2 (Brand 6)	0.69 ± 0.07^ g^	1.70 ± 0.07^f^	2.50 ± 0.07^e^	3.10 ± 0.14^d^	3.68 ± 0.14^c^	4.29 ± 0.07^b^	4.89 ± 0.07^a^
Sweet biscuit (Brand 7)	0.50 ± 0.07^ g^	1.19 ± 0.00^f^	1.59 ± 0.28^e^	1.88 ± 0.07^d^	2.50 ± 0.21^c^	3.00 ± 0.00^b^	3.60 ± 0.00^a^
Sweet-salty Biscuit (Brand 4)	0.69 ± 0.07^ g^	1.30 ± 0.07^f^	1.90 ± 0.07^e^	2.45 ± 0.07^d^	2.89 ± 0.14^c^	3.20 ± 0.00^b^	3.86 ± 0.14^a^
Sweet-salty biscuit (Brand 6)	0.65 ± 0.04^ g^	1.10 ± 0.07^f^	1.49 ± 0.07^e^	1.99 ± 0.07^d^	2.69 ± 0.14^c^	3.04 ± 0.04^b^	3.70 ± 0.07^a^
Salty biscuit (Brand 1)	0.50 ± 0.07^ g^	1.10 ± 0.07^f^	1.75 ± 0.04^e^	2.40 ± 0.14^d^	2.70 ± 0.14^c^	3.10 ± 0.14^b^	3.80 ± 0.28^a^
Atta bakery packaged biscuit	0.70 ± 0.07^ g^	1.20 ± 0.14^f^	1.90 ± 0.07^e^	2.49 ± 0.07^d^	3.09 ± 0.07^c^	3.74 ± 0.04^b^	4.29 ± 0.07^a^
Atta bakery unpackaged biscuit	0.50 ± 0.07^ g^	1.15 ± 0.04^f^	1.55 ± 0.04^e^	1.90 ± 0.07^d^	2.45 ± 0.04^c^	2.89 ± 0.14^b^	3.44 ± 0.14^a^

Values are mean ± SD, (*n* = 3), Values having different superscripts from a, b, c to g are significantly different from each other day wise in rows.

At initial stage, the peroxide values in all samples of sweet cookies ranged between 0.45 ± 0.04 to 0.70 ± 0.07 meq/kg (Table 4). The values increased significantly during storage. After 90 days, the corresponding figures ranged between 3.20 ± 0.00 to 4.40 ± 0.28 meq/kg. The initial peroxide values of packaged and unpackaged salty cookies were recorded as 0.45 ± 0.04 and 0.50 ± 0.07 meq/kg, respectively. The corresponding values at final stage were 3.70 ± 0.07 and 4.29 ± 0.07 meq/kg, respectively. Similar trend was observed in the peroxide values of packaged and unpackaged sweet cookies.

**Table 4:** Peroxide value of cookies.

Bakery products	Peroxide value (meq/kg)						
	0 DAY	15 DAY	30 DAY	45 DAY	60 DAY	75 DAY	90 DAY
Sweet cookies (Brand 4)	0.65 ± 0.04^ g^	1.05 ± 0.04^f^	1.54 ± 0.04^e^	1.80 ± 0.00^d^	2.39 ± 0.00^c^	2.99 ± 0.28^b^	3.20 ± 0.00^a^
Sweet cookies (Brand 7)	0.70 ± 0.07^ g^	1.49 ± 0.14^f^	2.20 ± 0.28^e^	2.89 ± 0.07^d^	3.29 ± 0.04^c^	3.75 ± 0.28^b^	4.40 ± 0.28^a^
Sweet cookies (Brand 8)	0.60 ± 0.00^ g^	1.25 ± 0.04^f^	1.80 ± 0.14^e^	2.30 ± 0.07^d^	2.89 ± 0.14^c^	3.60 ± 0.00^b^	4.09 ± 0.07^a^
Sweet cookies (Brand 9)	0.45 ± 0.04^ g^	1.09 ± 0.07^f^	1.45 ± 0.04^e^	1.80 ± 0.14^d^	2.34 ± 0.04^c^	2.75 ± 0.04^b^	3.30 ± 0.07^a^
Sweet bakery packaged cookies	0.65 ± 0.04^ g^	1.10 ± 0.07^f^	1.75 ± 0.04^e^	2.08 ± 0.14^d^	2.40 ± 0.14^c^	2.99 ± 0.28^b^	3.60 ± 0.00^a^
Sweet bakery unpackaged cookies	0.50 ± 0.07^ g^	1.10 ± 0.07^f^	1.69 ± 0.07^e^	2.09 ± 0.07^d^	2.69 ± 0.07^c^	3.04 ± 0.04^b^	3.74 ± 0.04^a^
Salty bakery packaged cookies	0.45 ± 0.04^ g^	0.90 ± 0.07^f^	1.50 ± 0.07^e^	2.09 ± 0.07^d^	2.69 ± 0.14^c^	3.10 ± 0.14^b^	3.70 ± 0.07^a^
Salty bakery unpackaged cookies	0.50 ± 0.07^ g^	1.05 ± 0.04^f^	1.69 ± 0.07^e^	2.20 ± 0.14^d^	2.89 ± 0.14^c^	3.70 ± 0.07^b^	4.29 ± 0.07^a^

Values are mean ± SD, (*n* = 3), Values having different superscripts from a, b, c to g are significantly different from each other day wise in rows.

#### Free fatty acids

The free fatty acids in all the samples of biscuits increased significantly during storage (Table 5). At initial stage, the FFA in sweet biscuits ranged between 0.02 ± 0.04 to 0.04 ± 0.07 percent. The values in all the samples increased significantly during storage time. After 90 days, the corresponding figures ranged between 0.16 ± 0.04 to 0.17 ± 0.14 percent. In terms of salty biscuits, the initial FFA was recorded as 0.04 ± 0.07 percent and after 90 days, FFA reached to 0.16 ± 0.07 percent. In sweet-salty biscuits, the initial FFA in brand 4 was recorded as 0.03 ± 0.00 percent and after storage it increased to 0.16 ± 0.11 percent whereas the initial FFA in brand 6 was observed as 0.04 ± 0.07 percent and final FFA was 0.16 ± 0.04 percent. In packaged *atta* biscuit, the initial FFA and final FFA was observed as 0.03 ± 0.00 and 0.16 ± 0.07 percent, respectively. The corresponding figures in unpackaged *atta* biscuits were 0.03 ± 0.00 and 0.16 ± 0.07 percent, respectively.

**Table 5:** Free fatty acids of biscuits.

Bakery products	Free fatty acids (%)						
	0 DAY	15 DAY	30 DAY	45 DAY	60 DAY	75 DAY	90 DAY
Sweet biscuit (Brand 1)	0.04 ± 0.07^f^	0.07 ± 0.07^e^	0.10 ± 0.07^d^	0.11 ± 0.00^d^	0.13 ± 0.07^c^	0.15 ± 0.07^b^	0.16 ± 0.21^a^
Sweet biscuit 1 (Brand 3)	0.02 ± 0.04^f^	0.06 ± 0.04^e^	0.08 ± 0.00^d^	0.10 ± 0.07^c^	0.11 ± 0.00^c^	0.13 ± 0.14^b^	0.14 ± 0.14^a^
Sweet biscuit 2 (Brand 3)	0.03 ± 0.00^f^	0.06 ± 0.14^e^	0.08 ± 0.00^d^	0.10 ± 0.07^c^	0.11 ± 0.14^c^	0.13 ± 0.07^b^	0.16 ± 0.04^a^
Sweet biscuit 1 (Brand 4)	0.03 ± 0.00^f^	0.07 ± 0.07^e^	0.09 ± 0.00^d^	0.10 ± 0.07^d^	0.13 ± 0.07^c^	0.14 ± 0.00^b^	0.16 ± 0.04^a^
Sweet biscuit 2 (Brand 4)	0.04 ± 0.07^e^	0.07 ± 0.07^d^	0.08 ± 0.11^d^	0.10 ± 0.00^c^	0.11 ± 0.14^c^	0.13 ± 0.07^b^	0.17 ± 0.14^a^
Sweet biscuit (Brand 5)	0.04 ± 0.07^f^	0.07 ± 0.07^e^	0.08 ± 0.00^d^	0.10 ± 0.07^c^	0.11 ± 0.14^c^	0.13 ± 0.18^b^	0.16 ± 0.04^a^
Sweet biscuit 1 (Brand 6)	0.02 ± 0.04^f^	0.06 ± 0.04^e^	0.08 ± 0.04^d^	0.10 ± 0.07^c^	0.11 ± 0.14^c^	0.13 ± 0.07^b^	0.16 ± 0.04^a^
Sweet biscuit 2 (Brand 6)	0.04 ± 0.07^f^	0.07 ± 0.07^e^	0.09 ± 0.04^d^	0.10 ± 0.07^d^	0.12 ± 0.04^c^	0.14 ± 0.07^b^	0.16 ± 0.07^a^
Sweet biscuit (Brand 7)	0.04 ± 0.04^f^	0.07 ± 0.07^e^	0.08 ± 0.04^e^	0.10 ± 0.07^d^	0.13 ± 0.07^c^	0.15 ± 0.04^b^	0.17 ± 0.00^a^
Sweet-salty Biscuit (Brand 4)	0.03 ± 0.00^ g^	0.06 ± 0.14^f^	0.08 ± 0.00^e^	0.09 ± 0.00^d^	0.11 ± 0.14^c^	0.13 ± 0.07^b^	0.16 ± 0.11^a^
Sweet-salty Biscuit (Brand 6)	0.04 ± 0.07^ g^	0.08 ± 0.00^f^	0.09 ± 0.00^e^	0.11 ± 0.14^d^	0.13 ± 0.07^c^	0.14 ± 0.00^b^	0.16 ± 0.04^a^
Salty biscuit (Brand 1)	0.04 ± 0.07^f^	0.06 ± 0.00^e^	0.08 ± 0.14^d^	0.10 ± 0.07^c^	0.11 ± 0.04^c^	0.13 ± 0.07^b^	0.16 ± 0.07^a^
Atta bakery packaged biscuit	0.03 ± 0.00^f^	0.04 ± 0.07^e^	0.07 ± 0.07^d^	0.08 ± 0.14^d^	0.10 ± 0.07^c^	0.13 ± 0.07^b^	0.16 ± 0.07^a^
Atta bakery unpackaged biscuit	0.03 ± 0.00^f^	0.06 ± 0.14^e^	0.08 ± 0.04^d^	0.10 ± 0.07^c^	0.11 ± 0.14^c^	0.13 ± 0.07^b^	0.16 ± 0.07^a^

Values are mean ± SD, (*n* = 3), Values having different superscripts from a, b, c to g are significantly different from each other day wise in rows.

At initial stage, the free fatty acids in all samples of sweet cookies ranged between 0.02 ± 0.04 to 0.04 ± 0.07 percent (Table 6). The values increased significantly during storage. After 90 days, the corresponding figures ranged between 0.15 ± 0.04 to 0.17 ± 0.11 percent. The free fatty acids of packaged and unpackaged salty cookies were recorded as 0.05 ± 0.04 and 0.03 ± 0.00 percent, respectively. The corresponding values at final stage were 0.31 ± 0.42 and 0.15 ± 0.11 percent, respectively. Similar trend was observed in the free fatty acids of packaged and unpackaged sweet cookies.

**Table 6:** Free fatty acids of cookies.

Bakery products	Free fatty acids (%)						
	0 DAY	15 DAY	30 DAY	45 DAY	60 DAY	75 DAY	90 DAY
Sweet cookies (Brand 4)	0.04 ± 0.07^e^	0.08 ± 0.11^d^	0.10 ± 0.07^c^	0.11 ± 0.07^c^	0.13 ± 0.07^b^	0.14 ± 0.00^b^	0.16 ± 0.07^a^
Sweet cookies (Brand 7)	0.05 ± 0.04^e^	0.07 ± 0.00^d^	0.10 ± 0.07^c^	0.11 ± 0.00^c^	0.12 ± 0.04^b^	0.14 ± 0.00^a^	0.15 ± 0.04^a^
Sweet cookies (Brand 8)	0.02 ± 0.04^ g^	0.05 ± 0.04^f^	0.08 ± 0.14^e^	0.10 ± 0.07^d^	0.13 ± 0.07^c^	0.15 ± 0.04^b^	0.17 ± 0.00^a^
Sweet cookies (Brand 9)	0.04 ± 0.04^e^	0.07 ± 0.07^d^	0.09 ± 0.04^c^	0.13 ± 0.07^b^	0.14 ± 0.14^b^	0.16 ± 0.21^a^	0.17 ± 0.11^a^
Sweet bakery packaged cookies	0.04 ± 0.00^e^	0.07 ± 0.07^d^	0.08 ± 0.00^d^	0.10 ± 0.07^c^	0.11 ± 0.00^c^	0.13 ± 0.11^b^	0.16 ± 0.11^a^
Sweet bakery unpackaged cookies	0.03 ± 0.00^e^	0.06 ± 0.14^d^	0.07 ± 0.07^d^	0.10 ± 0.07^c^	0.13 ± 0.07^b^	0.14 ± 0.00^b^	0.16 ± 0.07^a^
Salty bakery packaged cookies	0.05 ± 0.04^ g^	0.10 ± 0.07^f^	0.16 ± 0.04^e^	0.19 ± 0.11^d^	0.23 ± 0.07^c^	0.27 ± 0.07^b^	0.31 ± 0.42^a^
Salty bakery unpackaged cookies	0.03 ± 0.00^f^	0.04 ± 0.07^f^	0.07 ± 0.07^e^	0.09 ± 0.14^d^	0.11 ± 0.18^c^	0.13 ± 0.07^b^	0.15 ± 0.11^a^

Values are mean ± SD, (*n* = 3), Values having different superscripts from a, b, c to g are significantly different from each other day wise in rows.

## Discussion

This current study opens prospects for the bakery industry in India to utilize healthy fats for the sake of consumer’s health. In this study, researchers have analyzed two products of biscuit family that are biscuit and cookies. According to the NOVA categorization system, biscuits can be categorised as processed or ultra-processed. If they are made with culinary components, they are referred to as minimally processed foods. If these items do, however, contain ingredients like starch, gluten, lactose, vegetable oil, and/or food additives, they are regarded as ultra-processed^22^. The majority of companies in supermarkets produce ultra-processed products to draw in more customers and to stimulate overconsumption as these are made with intense flavours, sugar, salt, and fat^1,23^. Many studies witnessed that diets containing a greater amount of ultra-processed food had been considered poor in terms of nutritional quality^12,25,26,30,34^.

Biscuits can be wonderful options for consumers looking for taste and convenience because they are typically offered in individual packets, are foods that are ready to eat, are convenient to carry around or store at home, and have a cheap price^15^. However, the majority of biscuits are highly processed, showing that it can be challenging for shoppers to choose wisely at supermarkets^18^. Additionally, fat is a crucial component for giving the biscuit its distinctive soft and crunchy texture. Investigating the kind of fat present in frequently eaten items is therefore critical because diet-related fatty acid composition has been linked to a variety of cardiovascular illnesses^8,21^.

The findings of current research work revealed, vegetable fat was present in most biscuits and cookies. The fatty acid composition of this fat was variable, depending on its origin. It was observed that SFAs were more common and palmitic acid was the major SFA found in all the biscuits. This might be due to the use of palm oil in formulation of biscuits. Dias et al.^11^ also observed that palmitic acid was the most prevalent fatty acid in 14 out of 19 different types of biscuits. One previous study reported that depending on the type of biscuit, the total content of SFA ranged from 14.8 to 60 percent, MUFA from 32.4 to 57.5 percent, and PUFA from 5.8 to 26.8 percent^28^. According to Amrutha Kala ^3^, the amount of SFA, MUFA and PUFA ranged between 5.1–18.7 g, 0.9–8.6 g and 0.2–3.5 g per 100 g, respectively. Similarly, palmitic acid was also the majorly observed SFA in all the cookies. Similarly, Trattner et al. ^33^ also found that the percentage of SFA and PUFA increased due to high percentage of palmitic acid and linoleic acid, respectively. The fact that most biscuits were sweet demonstrates both the industry's and consumers' interest in these goods. When compared to salty biscuits, the selected sweet biscuits in the present study had more total fat and saturated fat, giving them high caloric density. The over consumption of sugar and fat has been associated with many health related problems such as obesity, diabetes, hepatic steatosis, and other chronic diseases^13,19^.

Further, MUFA made up the majority of the unsaturated fatty acids in the selected goods. Earlier studies have documented that lower concentration of PUFA (especially linolenic acid, C18:3) and higher concentration content of MUFA (e.g. mainly oleic acid, C18:1 9c) provide higher oil stability^36^. Therefore, the end product may be used whenever foods need to be cooked or fired at high temperatures. MUFA rich products have also been found less prone to oxidation as compared to PUFA rich food products^29^. In the present work, oxidative stability was measured through the analysis of PV and FFA. The reactive oxygen contents expressed in terms of milliequivalents (meq) of free iodine per kg of fat is known as PV. It is calculated by titrating potassium iodide-released iodine with sodium thiosulphate solution^6^. Triacylglycerol is converted into FFA through the cleavage of ester bonds by the actions of lipase, high temperatures, and moisture. FFA is frequently used to describe the oil's quality and suitability for use in food. It is important to know that how much the oxidative stability in term of PV and FFA has changed throughout shelf life of these products for consumer health and product quality. This is because long storage time may be involved between preparation and consumption of the foodstuff^9^**.**

In the current study, the increase in the value of free fatty acid and peroxide values of the bakery products were observed during storage. But, the PV of all the products even after storage period of three months was found below the permissible limits (< 10 meq/kg) as recommended by FSSAI (2021). Calligaris et al.^6^ also studied the peroxide value of biscuit stored at different temperatures (-18^0^C to 45^0^C) for 160 days. The study showed that the peroxide values also increased as the storage time increased. Similarly, FFA value of all the products did not cross acceptable level of 0.5 percent as given by FSSAI (2021). Manzocco et al.^20^ stated that shelf life of the product was dependent on the oxidative stability of the fat components. Reshma et al.^27^ also observed that biscuit samples had the least free fatty acid value i.e., 0.52 ± 0.20 percent.

## Conclusion

The present study reported fatty acid profiling of 22 bakery products of biscuit family belonging to two major categories, viz., packaged and unpackaged. Nine detected fatty acids were classified into three categories SFA, MUFA and PUFA, of which SFA represented the most abundant class in all samples. Among SFAs, palmitic acid was the most abundant among all fatty acids in packaged and unpackaged samples. Out of total selected eight brands, six were national and two were international. It was observed that amount of palmitic acid was higher in the products belonging to local brands. This showed the reluctance amongst food manufacturers in India towards replacing SFA with MUFA or PUFA because of the issue related to cost and physical properties. Moreover, the selected products were mostly ultra-processed. The food guidelines of many countries have specified that ultra-processed food should be avoided, and priority should be given to *in natura* and minimally processed foods. Hence, it is crucial to use a variety of techniques today to limit the consumption of highly processed foods, which may also result in a decrease in the consumption of calories, saturated and trans fats, sugar, and/or sodium. The limitation of present study was that it did not analyze more details of the product such as other macronutirents and chemical food additives. Therefore, additional research is required to advance understanding of the topic.

## Data Availability

The data are available from the corresponding author upon reasonable request and with the permission from institution.
